# Reference materials for MS-based untargeted metabolomics and lipidomics: a review by the metabolomics quality assurance and quality control consortium (mQACC)

**DOI:** 10.1007/s11306-021-01848-6

**Published:** 2022-04-09

**Authors:** Katrice A. Lippa, Juan J. Aristizabal-Henao, Richard D. Beger, John A. Bowden, Corey Broeckling, Chris Beecher, W. Clay Davis, Warwick B. Dunn, Roberto Flores, Royston Goodacre, Gonçalo J. Gouveia, Amy C. Harms, Thomas Hartung, Christina M. Jones, Matthew R. Lewis, Ioanna Ntai, Andrew J. Percy, Dan Raftery, Tracey B. Schock, Jinchun Sun, Georgios Theodoridis, Fariba Tayyari, Federico Torta, Candice Z. Ulmer, Ian Wilson, Baljit K. Ubhi

**Affiliations:** 1 Chemical Sciences Division, National Institute of Standards and Technology (NIST), Gaithersburg, MD 20899 USA; 2 Department of Physiological Sciences, Center for Environmental and Human Toxicology, College of Veterinary Medicine, University of Florida, Gainesville, FL 32610 USA; 3 BERG LLC, 500 Old Connecticut Path, Building B, 3rd Floor, Framingham, MA 01710 USA; 4 Division of Systems Biology, National Center for Toxicological Research, U.S. Food and Drug Administration (FDA), Jefferson, AR 72079 USA; 5 Analytical Resources Core: Bioanalysis and Omics Center, Colorado State University, Fort Collins, CO 80523 USA; 6 IROA Technologies, Chapel Hill, NC 27517 USA; 7 Chemical Sciences Division, National Institute of Standards and Technology (NIST), Charleston, SC 29412 USA; 8 School of Biosciences, Institute of Metabolism and Systems Research and Phenome Centre Birmingham, University of Birmingham, Birmingham, B15, 2TT UK; 9 Division of Program Coordination, Planning and Strategic Initiatives, Office of Nutrition Research, Office of the Director, National Institutes of Health (NIH), Bethesda, MD 20892 USA; 10 Department of Biochemistry and Systems Biology, Institute of Systems, Molecular and Integrative Biology, University of Liverpool, BioSciences Building, Crown St., Liverpool, L69 7ZB UK; 11 Department of Biochemistry and Molecular Biology, University of Georgia, Athens, GA 30602 USA; 12 Biomedical Metabolomics Facility Leiden, Leiden University, Einsteinweg 55, 2333 CC Leiden, The Netherlands; 13 Bloomberg School of Public Health, Environmental Health and Engineering, Johns Hopkins University, Baltimore, MD 21205 USA; 14 National Phenome Centre, Imperial College London, London, SW7 2AZ UK; 15 Thermo Fisher Scientific, San Jose, CA 95134 USA; 16 Cambridge Isotope Laboratories, Inc., Tewksbury, MA 01876 USA; 17 Northwest Metabolomics Research Center, University of Washington, Seattle, WA 98109 USA; 18 Department of Chemistry, Aristotle University, 54124 Thessaloniki, Greece; 19 Department of Internal Medicine, University of Iowa, Iowa City, IA 52242 USA; 20 Centre for Life Sciences, National University of Singapore, 28 Medical Drive, Singapore, 117456 Singapore; 21 Centers for Disease Control and Prevention (CDC), Atlanta, GA 30341 USA; 22 Computational & Systems Medicine, Imperial College, Exhibition Rd, London, SW7 2AZ UK; 23 MOBILion Systems Inc., 4 Hillman Drive Suite 130, Chadds Ford, PA 19317 USA

**Keywords:** Reference materials, Certified reference materials, Internal standards, Untargeted analysis, Mass spectrometry, Metabolomics, Lipidomics, Metabolomics quality assurance and quality control consortium (mQACC)

## Abstract

**Introduction:**

The metabolomics quality assurance and quality control consortium (mQACC) is enabling the identification, development, prioritization, and promotion of suitable reference materials (RMs) to be used in quality assurance (QA) and quality control (QC) for untargeted metabolomics research.

**Objectives:**

This review aims to highlight current RMs, and methodologies used within untargeted metabolomics and lipidomics communities to ensure standardization of results obtained from data analysis, interpretation and cross-study, and cross-laboratory comparisons. The essence of the aims is also applicable to other ‘omics areas that generate high dimensional data.

**Results:**

The potential for game-changing biochemical discoveries through mass spectrometry-based (MS) untargeted metabolomics and lipidomics are predicated on the evolution of more confident qualitative (and eventually quantitative) results from research laboratories. RMs are thus critical QC tools to be able to assure standardization, comparability, repeatability and reproducibility for untargeted data analysis, interpretation, to compare data within and across studies and across multiple laboratories. Standard operating procedures (SOPs) that promote, describe and exemplify the use of RMs will also improve QC for the metabolomics and lipidomics communities.

**Conclusions:**

The application of RMs described in this review may significantly improve data quality to support metabolomics and lipidomics research. The continued development and deployment of new RMs, together with interlaboratory studies and educational outreach and training, will further promote sound QA practices in the community.

## Introduction

The metabolomics Quality Assurance and Quality Control Consortium (mQACC) was established in 2018 to build a collaborative effort among relevant stakeholders from academia, industry, and governmental organizations to address key quality assurance (QA) and quality control (QC) issues in untargeted metabolomics (Beger et al., 2019). As part of its mission, the mQACC is engaging the metabolomics community to identify and to prioritize key reference materials (RMs) to be used in QA/QC for untargeted metabolomics research. RMs are artifact-based measurement standards that have been characterized for a known composition of specific physical, chemical or biological properties. They are often described by their function (e.g., calibration, quality control, method validation) and range in design from matrix-based materials from natural (e.g., biological) sources to “matrix-free” standards, such as pure substances or standard solutions and mixtures. The focus of untargeted metabolomics research is to detect and identify hundreds of metabolites and minimize sources of variance (biological *versus* technical) to identify differential metabolomics patterns of interest with an eventual goal to quantify select metabolites of biological interest. Thus, the appropriate use of RMs in untargeted metabolomics applications will provide confidence for such measurements and data standardization methods from different instrumental platforms, thereby ensuring suitable translation of biological discoveries through the elucidation of biomarkers or understanding of biological mechanisms.

Technological advances have allowed mass spectrometry (MS)-based untargeted metabolomics and lipidomics to be widely adopted in research laboratories. In addition to pushing the boundaries of biochemical research, including translational and precision medicine, untargeted analyses contribute to the advancement of nutritional assessment, fermentative optimizations, and agricultural productivity. Given its predominant usage in the metabolomics and lipidomics communities, MS hyphenated to chromatography separation techniques (e.g., liquid or gas chromatography with MS-based detection, LC–MS or GC–MS) represent a primary analytical method for untargeted metabolomics but also present unique challenges. The mQACC has recently defined the specific measurement challenges that different types of RMs may potentially pose and address best use practices for RMs by the metabolomics community (Evans et al., 2020). This effort directly builds upon prior considerations (Bowden et al., 2018; Broadhurst et al., 2018; Dudzik et al., 2018; Ribbenstedt et al., 2018; Schrimpe-Rutledge et al., 2016; Viant et al., 2019) of the analytical and QA/QC challenges faced in MS-based untargeted metabolomics and lipidomics, in contrast to the more common targeted metabolomics approaches. More recently, Alseekh et al. (2021) describe practical considerations for MS-based metabolomic workflows to improve the quality and comparability of resultant data and metadata. All of these efforts aim to demonstrate, disseminate and promote QA procedures and QC reference materials to be used across the community and enable metabolomics and lipidomics researchers to quickly adopt such practices to ultimately produce high-quality data and results.

QA/QC is critical to ensure that quality results are obtained from the diverse range of chromatographic separation approaches and MS-based detection methods that exist across laboratories. This diversity is due in part to available instrumentation, available processing software, the specific goals of the project, and the sample types used in the specific studies. Effective QA/QC in untargeted metabolomics requires the interplay between the two quality management processes (Broadhurst et al., 2018). QA is considered the processes that ensures quality results before actual measurements are conducted, such as the development and use of Standard Operating Procedures (SOPs) with corresponding training of metabolomics researchers and personnel. QC is the day-to-day operational techniques and processes of evaluating the quality of results and overall laboratory performance, which often includes the use of RMs. While current RMs cannot be expected to immediately solve all QC issues associated with MS-based untargeted metabolomics, they can be used in developing future approaches towards more confident compound identification (Levels 1 and 2 of the Metabolomics Standards Initiative) (Sumner et al., 2007), increased reproducibility of results and eventual quantification, while also acting as a necessary bridge for comparability of results across multiple analytical platforms (including nuclear magnetic resonance spectroscopy (NMR)-based untargeted metabolomics) and among other laboratories.

Accordingly, the mQACC is actively working with the broader metabolomics community to develop measurement designs, protocols, and methods together with supporting materials comprised of solution-based and matrix-relevant RMs that can be utilized across instrumentation platforms for routine QA/QC practices in untargeted metabolomics. The development of unified products that include associated reference data are a future goal and will be essential QA/QC tools for fully confident results to be obtained from untargeted metabolomics and lipidomics studies. The development of many of these RMs is being spearheaded by commercial organizations and government agencies in direct response to a community need, facilitating broader distribution among international research communities, which serves as a framework for increasingly coordinated generation of standardized and quality-controlled data.

Munafò et al. (2017) have highlighted that there is an urgent need to increase reproducibility in science, and that part of the problem is the lack of transparency in reporting studies. Therefore, having confidence in the data generated will reduce uncertainty within experimental pipelines and therefore improve laboratory performance and standardization across laboratories. For metabolomics, and indeed all the ‘omics, transparency in data is central to this. The provision of open data accompanying any reported studies should be first encouraged and then expected. These freely available data should be readily interpretable and include the metabolomics data on the samples themselves, along with any associated metadata about these samples, as well as data on the QA and the QC samples that have been analyzed as part of the metabolomics pipeline.

Originating from its nonregulatory objectives, the mQACC consortium aims to present all potential QA/QC solutions that can improve overall laboratory performance and facilitate more comparable metabolomics and lipidomics measurements across the community, while maintaining neutrality to all potential products. For this review, the products are principally MS-based application centric and include commercial reference standard producers, RMs from government organizations, and any associated reference data sources from commercial entities, academic institutions and/or other organizations. In each subsequent section, we will review how methodologies have emerged and evolved to the current state of the art, concluding with a look at what promising developments are on the horizon to further enable QC and drive data transparency and reproducibility, as well as integrity and confidence in untargeted MS-based metabolomics studies, both within and across laboratories.

## Reference materials for untargeted metabolomics

The progression of metabolomics and lipidomics research to meet the ever-increasing demands of continuity and scale driven by large single and multi-site clinical and epidemiological studies, has created a need to demonstrate confidence in analytical performance. The same is true of other metabolomics and lipidomics research, where many thousands of samples may be analyzed; for example, for predicting gene-phenotype links in large-scale functional genomics studies or in plant breeding. One of the priority efforts of the mQACC is to envision the key characteristics for broadly applicable and sustainable RMs that the community can afford. An initial effort has focused on blood- and urine-based RMs, but other types of materials including (but not limited to) synthetic mixtures, endogenous materials, spiked and isotopically-labeled materials (including from bacterial, yeast or eukaryotic cell culture), disease- and species-specific materials, and various tissues (including from plants) with their associated extracts are an ultimate goal. Together with development of RMs, a consideration of what type of associated reference data (and metadata) for assessing metabolomics data quality is a critical parallel effort.

In a recent study (Evans et al., 2020), it was determined that only 33% of metabolomics laboratories use RMs regularly and that the use of RMs was not consistent across individual laboratories; some laboratories use RMs as a long-term reference QC samples, whereas others utilize them for cross-platform evaluations or interlaboratory studies. Similarly, a survey from over 125 laboratories in the lipidomics community (Bowden et al., 2018) indicated that a wide methodological diversity exists; less than half of laboratories formally establish and adhere to SOPs and QC practices. Further, most of the laboratories do not have standardized policies for the adoption of methods and protocols, including the use of measurement standards, software, and quantification procedures, and reporting of false positive results in lipid identification.

### Definitions

Towards the development of solutions for improving QA/QC in untargeted metabolomics, the use of RMs aim to address overcome potential QC barriers in the various analytical and data acquisition steps. As previously introduced, the general concept of RMs can be broad, and the various forms of RMs are utilized for a range of applications (see Table 1). RMs include Certified Reference Materials (CRMs) (highly characterized RMs supplied with a certificate of analysis), synthetic reference standards, solutions and standard mixtures, and Reference Library (RL) products that are also comprised of higher purity standards. Often referred to as pooled QC samples, QCRMs can be study specific (Bijlsma et al., 2006; Sangster et al., 2006) or study independent (i.e. surrogate) (Dunn et al., 2011a, 2011b) including those intended for longer term use, for example across multiple studies and/or platforms within or across laboratories (termed “Long term Reference” or LTR samples) (Lewis et al., 2016). Each also include long-term reference QC samples (Broadhurst et al., 2018) and can be operationally defined to serve in a similar capacity to a RM for control and reporting of observed variation in untargeted profiling measurements. Other important QC samples include extraction or process blanks and system conditioning samples, but these are not considered RMs in a general sense. RMs have a range of applications as are described in this review and can be applied towards pre- or post-sample processing steps, including modifying instrument acquisition and MS-tuning parameters (Bouhifd et al., 2015) or the development of post-acquisition informatic approaches.

**Table 1:** Definitions of RMs and CRMs, and descriptions of other measurement standards commonly employed for QC in untargeted metabolomics

Category	ISO definitions (ISO, 2016) and descriptions	Practical usage
Reference material (RM)	17,034 Sect. 3.3 Definition: A material, sufficiently homogeneous and stable with respect to one or more specified properties, which has been established to be fit for its intended use in a measurement process Notes: Reference material is a generic term. Properties can be quantitative or qualitative, e.g. identity of substances or species. Uses may include the calibration of a measurement system, assessment of a measurement procedure, assigning values to other materials, and quality control	RM is a generic term and is generally used to describe a wide range of materials and measurement standards used in QC
Certified reference material (CRM)	17,034 Sect. 3.2 Definition: A reference material characterized by a metrologically valid procedure for one or more specified properties, accompanied by a reference material certificate that provides the value of the specified property, its associated uncertainty, and a statement of metrological traceability Notes: The concept of value includes a nominal property or a qualitative attribute such as identity or sequence. Uncertainties for such attributes may be expressed as probabilities or levels of confidence	CRMs are considered within the broader definition of RMs. CRMs are highly specialized materials, which are generally only produced in a few highly specific areas, where critical measurement requirements and traceability considerations must be met In practice, pure chemical and solution CRMs are designed for calibration, chemical identification and SI traceability. Matrix-based CRMs are designed for method validation and accuracy control applications, but can also be used for intra-laboratory QC, interlaboratory assessments and method harmonization efforts
Quality control reference material (QCRM)	Description: Pooled materials comprised of subsets from all (or a representative subset) of the biological test samples in a specific study, that are well mixed into a homogenous pool and then aliquoted into subsamples. (Often termed pooled QC materials.)	QCRMs are used for general QC measurements for the study of origin but are also well suited to intra‑laboratory assessment or routine analysis. They can also be utilized in other quality assurance purposes, such as quality management system training. Can also be repurposed for interlaboratory method harmonization
Standard mixtures	Description: Mixtures of reference standards of pure chemical compounds in a homogenous solution form that have been well characterized	Even though these standards are usually prepared for use as calibration standards for quantitative (targeted) analysis, they can be used in chemical identification for untargeted approaches. Standard mixtures are generally prepared with enough aliquots to be widely available and to be stable for a sufficient period of time
Reference library (RL)	Description: Collections of pure authenticated compounds prepared either in neat form or as individual solutions or as defined standard mixtures	The compounds and/or mixtures are used for chemical identification or system suitability applications. Often the individual chemical standards are provided initially as lyophilized (dried) to be reconstituted in an appropriate solvent

The terms RMs and CRMs are used throughout the various fields of analytics and bio-analytics and are explicitly defined by the International Organization for Standardization (ISO) (ISO, 2016). The purpose, intended use and scope of RMs and CRMs are also described in ISO Standard 17,034 (ISO, 2021) and its related Standards and Guides (Trapmann et al., 2017). Table 1 summarizes these definitions.

A measurement standard (and its derived term reference measurement standard) is the embodiment of the realization of a given quantity (with a stated value and associated measurement uncertainty) that is used as a reference (JCGM, 2012). This term is often used synonymously with calibrator and implies the determination of quantitative values through a calibration measurement procedure. Many reference measurement standards (i.e., reference standards) are utilized in quantitative determinations for targeted metabolomics; however, for untargeted metabolomics, these standards are used largely for chemical identification purposes.

### Design considerations

RMs are designed to be stable and homogenous, ideally with long-term stability with respect to the components of interest under defined storage conditions. Matrix-based RMs should also be designed with appropriate matrices for the intended QC applications. Further guidance on RMs in the context of alternatives to animal tests (Hoffmann et al., 2008) can be easily translated to the case for RMs for untargeted metabolomics and lipidomics. The characterization of these materials should include reliable and confident chemical identity, mass concentrations (when relevant) and metrological traceability of specific components. The characterization should also include descriptions of relevant impurities, physico-chemical properties, use by dates, and safety considerations associated with their typical usage. The availability of high-quality reference results associated with typical laboratory usage would be highly desirable; this would include the adequate potency within response range of evaluation, such as detection limit and dynamic range of the instrumental platform, but also the historical performance and reproducibility of results. Universal access to these materials and their affordability (i.e., cost) should be a goal in production through suitable commercial vendors and private and governmental organizations. Furthermore, the resistance to the overall expense of these products and the limited guidance and “best practices” available on how to utilize them effectively also presents a challenge for widespread acceptance within the metabolomics and lipidomics communities. For standard mixtures of authentic standards as (certified) reference materials, composition, purity, and concentration are readily available, often supported by a certificate of analysis and easily reported. In the case of biological matrices (e.g., the 1950 plasma provided by NIST) many of these features are less readily available as such mixtures are complex and incompletely characterized in terms of all their constituents. Arguably, biological matrices not really qualify as CRM and especially QCRM as the composition and stability of components cannot be defined. However, pragmatically, often no more traceable mixtures can be created, but caution is advised that such ill-defined RM can serve as CRM or QCRM only for defined properties.

Note: while measurement reproducibility has a formal metrological definition (JCGM, 2012), herein it is used to mean that measurements on similar materials made by different laboratories and/or at different times obtain similar results.

As part of the development process, RMs are designed to be fit-for-purpose as documented by their intended usage statements (ISO, 2017) and thus appropriate RMs can be used to harmonize results obtained by individual instruments and procedures and directly compared to the reference results. As they are ideally commercially available, the more widely such RMs are used, the greater the potential exists for the comparability and harmonization of results across laboratories and over time. As a pooled matrix-based (e.g., serum, plasma, urine) RM becomes adopted and widely used, it is inevitable that the initial production batch will become depleted. Thus, the challenge exists for RM producers to replenish with contiguous replacement materials that maintain suitable characteristics for use in existing nontargeted metabolomics QC applications. Notably, as RMs become more specific in both how they are designed and characterized for intended usage (e.g., sample type and form), the likelihood that they become universally adopted is reduced. A limited selection of appropriate matrix-matched RMs for specific metabolomics and lipidomics applications remains an ever-present limitation.

### Usage

**Fig. 1:**
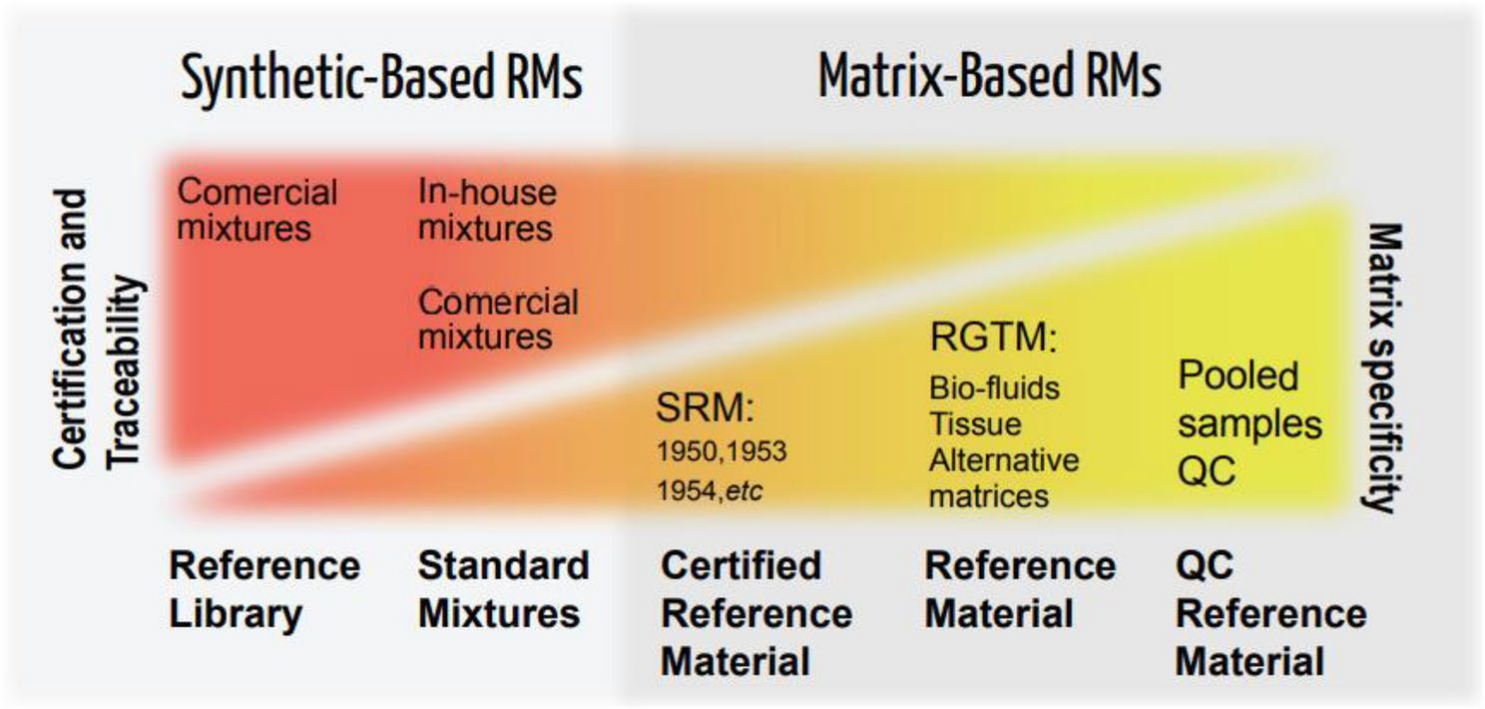
Range of reference materials employed in the field of metabolomics and lipidomics. Gradient colors from yellow to red represent the inverse relationship between matrix specificity to the study samples and the metabolite traceability to certified standards. Each of these RMs can be applied to capture the inherent unwanted technical variance across the numerous steps that make up a metabolomics workflow. RM assessment is to be carried out before, during and after in accordance with the defined best practices and QA/QC system

An example of the range of reference materials employed in the field of metabolomics and lipidomics is provided in Fig. 1. RMs can be leveraged to determine and reduce measurement variability associated with the analytical sample preparation (and some select pre-analytical controls), instrumental analysis and system suitability, compound identification, and data processing and analysis steps for which the standards have been qualified. A further example (Evans et al., 2020) depicts the analysis of RMs as quality control (in addition to other QC tools) within a continuum of QC practices. The development and use of SOPs for a RM would fall under QA practices that comprise an overarching quality management system.

Routine usage of RMs with associated QA and QC protocols benefits not only the quality of their data for individual laboratories, but it also enhances the entire field with increased comparability of results and conclusions amongst laboratories and individual studies, which will lead to more open data and robustness and reproducibility for metabolomics and lipidomics. However, some analytical method variation across laboratories with respect to metabolite identification and/or profiles is still to be expected for these reference materials as a result of differences in procedures and instrumentation.

However, the wide adoption of RMs will increase our understanding of the sources of variation and help to minimize bias between method results. As an example, retention order can change using different column chemistry and/or mobile phase composition; Vaughan et al. discuss the application of contiguous samples run in 100 different laboratories using 100 different analytical methods and has generated a comparable dataset suitable for developing a widely useful retention time prediction model (Vaughan et al., 2012). It is also known that the use of different mass spectrometers can result in different relative intensities for the same metabolites, which can lead to differences in the results of multivariate analysis (Gika et al., 2010).

Use of a common RM in two datasets can serve as a key to aligning signals across an otherwise disparate sets of samples. The exact composition of the RM (e.g., a synthetic chemical standard mixture or a matrix-matched RM) will dictate its utility in alignment within the chromatographic retention time, *m*/*z*, and signal intensity (i.e., response factor) dimensions and the extent of its applicability to the global profile. Where successful, datasets aligned using a common RM can enable a higher degree of comparability and increased statistical power leading to more confidence in the biological knowledge gleaned from combined studies. Results obtained from a collective set are more extensible than that of the single study and may enable new questions to be asked of the data which were not anticipated in the original design. An important requirement for such an approach is that each sample set has clearly defined data and metadata organized consistent with the FAIR guidelines—Findable, Accessible, Interoperable, and Reproducible (Wilkinson et al., 2016). These guidelines do not act as a standard or specification as such, but rather as guiding principles for the reporting of data and metadata recently exemplified in system biology, drug discovery and other biomedical fields that can benefit from data reusability and hence further knowledge discovery.

## Reference materials of biological origin

Biological reference materials are RMs with biological (rather than synthetic) origin that are characterized for specific biological or chemical properties and frequently serve as QC materials to support numerous QC practices including: assessment of analytical system suitability, evaluation of measurement reproducibility, and fusion of batched data. They are often pooled materials and designed to be representative of the natural biochemical complexity observed in a given sample type (e.g., biofluid or tissue). These RMs address a different need than spike-in QC materials added for the evaluation samples at a specific stage during processing or to pooled QC materials that are applied in each analytical evaluation (Broadhurst et al., 2018). In recent years, the use of biological RMs with defined SOPs has become a cornerstone of metabolomics and lipidomics profiling interlaboratory studies, allowing the highly complex data generated to be compared across numerous sites to either generate (untargeted studies) or answer (targeted studies) hypotheses. Use of biological RMs and deposition of the resulting data to repositories also enable multi-laboratory data compilations and benchmarking of community practices and measurement methodologies for advancing intra- and interlaboratory harmonization.

### NIST SRM 1950

One of the first CRMs designed specifically for targeted metabolomics and made widely available to the research community was the National Institute of Standards and Technology (NIST) Standard Reference Material® (SRM) 1950 Metabolites in Frozen Human Plasma. This CRM was designed as a “universal matrix” to include plasma from 100 individuals from an equal number of men and women in a narrow adult age range (40–50 years) together with a racial distribution of the donors reflecting the distribution in the US population at the time of implementation. The CRM is specifically intended for the validation of analytical methods used in the determination of various nutritional and health status markers for clinically relevant metabolites in human plasma and similar materials. It has been value-assigned for nearly 100 electrolytes, amino acids, vitamins, hormones, and fatty acids (Phinney et al., 2013; Simón-Manso et al., 2013). SRM 1950 has since been used to validate novel measurement methodologies, protocols and workflows for metabolomics and lipidomics in the profiling and quantification of targeted compounds (Azab et al., 2019; Colas et al., 2014; Hermann et al., 2018; Lange & Fedorova, 2020; Misra & Olivier, 2020; Rampler et al., 2018; Rathod et al., 2020; Ribbenstedt et al., 2018; Roy et al., 2016; Schoeny et al., 2020; Schwaiger et al., 2018; Triebl et al., 2020; Ulmer et al., 2018; Wang et al., 2018). It has also recently been used for comparing methods, platforms, and data analysis for untargeted metabolomics and lipidomics applications (Cajka et al., 2017; Di Giovanni et al., 2020; Drotleff et al., 2019; Koelmel et al., 2020; Nichols et al., 2018; Ribbenstedt et al., 2018; Telu et al., 2016).

Many researchers performing untargeted metabolomics and lipidomics have adopted SRM 1950 as a long-term reference QC sample. It has been employed in evaluating instrumental performance, correction for batch variance, and ensuring comparability within and across laboratory studies (Liu et al., 2020). The extension of SRM 1950 as a control material for lipidomics evaluations is more recent (Aristizabal-Henao et al., 2020) and has been fundamental for the development of the human plasma lipidomic field. In 2010, Quehenberger et al. (2010) published a semi-quantitative description of SRM 1950, reporting the levels of over 500 distinct molecular species distributed among the major lipid classes. In this work, supported by the LIPID MAPS initiative, SRM 1950 promoted the use of adequate analytical methodologies to quantify the large spectrum of plasma lipids. The multiple targeted approaches adopted to generate the results were based on multiple reaction monitoring (MRM) detection and could almost cover the same number of species normally reported in untargeted experiments after validation of features.

The interest in the use of biological matrix-based RMs in the lipidomic community was furthered with the inclusion of SRM 1950 as a common QC material in a recent interlaboratory study that generated both lipid identifications and quantitative and/or semi-quantitative estimates of lipid concentrations (Bowden et al., 2017). While primarily a targeted MS-based lipidomics effort, this study highlighted significant disparities in the lipid concentrations and profiles reported by the participants, possibly due to the use of different internal standards, extraction methods and MS techniques. This has furthered efforts to develop guidelines and harmonize lipidomic workflows (Bowden et al., 2018) for the entire community. In the meanwhile, lipids in SRM 1950 have been characterized and quantified by other metabolomics global initiatives and in more controlled conditions, as part of a comprehensive list of metabolites that were reported in these efforts (Koelmel et al., 2020).

### Plasma and urine RM suites

SRM 1950 was originally designed as a standalone “normal” human plasma pooled material to validate analytical methods for targeted metabolite determinations rather than for untargeted profiling purposes. As this SRM contains nearly 50 measurands that are certified (and another 50 that are non-certified) by NIST, the cost remains relatively high, and is thus not practical for use as a routine and forever-sustained metabolomics QC material. NIST has thus recently established the Metabolomics Quality Assurance and Quality Control Materials (MetQual) program in an effort to improve the comparability of untargeted metabolomics measurements across all sectors, including industry, government, and academic laboratories, and provide access to new NIST RMs for metabolomics with a cost-friendly option. This includes the four-part candidate RM 8231 Frozen Human Plasma Suite for Metabolomics that can be used as QC materials for untargeted, differential metabolomics composed of pooled plasma: Part A. Diabetic Plasma, Part B. High Triglyceride Plasma, Part C. Young, African–American Plasma, and Part D. Normal Human Plasma from the same source as SRM 1950. This RM suite has been designed specifically for untargeted metabolomic analysis. Furthermore, the lipidomic profiles of the RM plasma suite have been characterized (Aristizabal-Henao et al., 2020), and its utility to benchmark the performance of data processing tools has been established (Riquelme et al., 2020).

A complementary suite of pooled urine RMs composed of both female and male smokers and non-smokers is also under development. These suites are intended to provide the metabolomics community with additional options to single-point QCRMs, such as SRM 1950. Moreover, in contrast to the extensive (and costly) value assignment of SRM 1950, these plasma and urine RM suites will be characterized by NIST and metabolomics stakeholders in through community-based interlaboratory studies. Characterization will include metabolite identification, annotation, fold changes and percent differences for purposes of underpinning differential metabolomics (Bearden et al., 2019) and lipidomics (Aristizabal-Henao et al., 2020) studies (see Sect. 6.1).

### Tissue-based reference materials

In contrast to metabolomics studies that employ common biofluids, direct analysis of a tissue provides a specific understanding of the biochemical function of a specialized organ or local environment. Such studies may seek to correlate the more invasive tissue diagnostic markers with profile signatures identified in biofluids. Reproducibility and QC practices are equally important for tissue research, and to the best of our knowledge, these practices rely on in-house developed pooled materials, a limitation to broader harmonization. To this end, NIST is developing tissue based RMs specifically for untargeted and differential based lipidomic, metabolomic and proteomic measurements in model and non-model species.

Research involving metabolomics of tissue commonly use liver, heart, kidney, skeletal muscle, or lungs, to name a few; however, with the complex process involving the design and production of RMs (Sect. 2.2), it is improbable to produce a RM for every common matrix. Challenges in RM development include acquiring large quantities of material that can be available to the community for upwards of a decade. Obtaining limited supply, human-sourced tissues amplifies this challenge. Furthermore, the time and expense of adding metrological traceability to the chemical characterization of these highly-complex matrix materials adds to the costs of RM production which are ultimately transferred to the customer.

In researching options for the first tissue-based omics material, NIST prioritized minimizing cost and production time and concentrated on a common study tissue. A fortunate opportunity presented itself with a collection of human livers cryogenically preserved and archived at the NIST Biorepository. The livers were preserved for a collaborative project between NIST and the Environmental Protection Agency (EPA) in 1979 to establish an environmental specimen banking system aimed at evaluating risks to human health and the environment due to the influx of man- made substances into the ecosystem. Though these tissues were originally collected for biomonitoring purposes, the goals of the NIST Biorepository have expanded to include the use of archived tissues for additional applications such as genetics, metabolomics, and proteomics. The livers are a source of several materials mentioned within this section.

#### Liver suite for untargeted metabolomics analysis

**Fig. 2:**
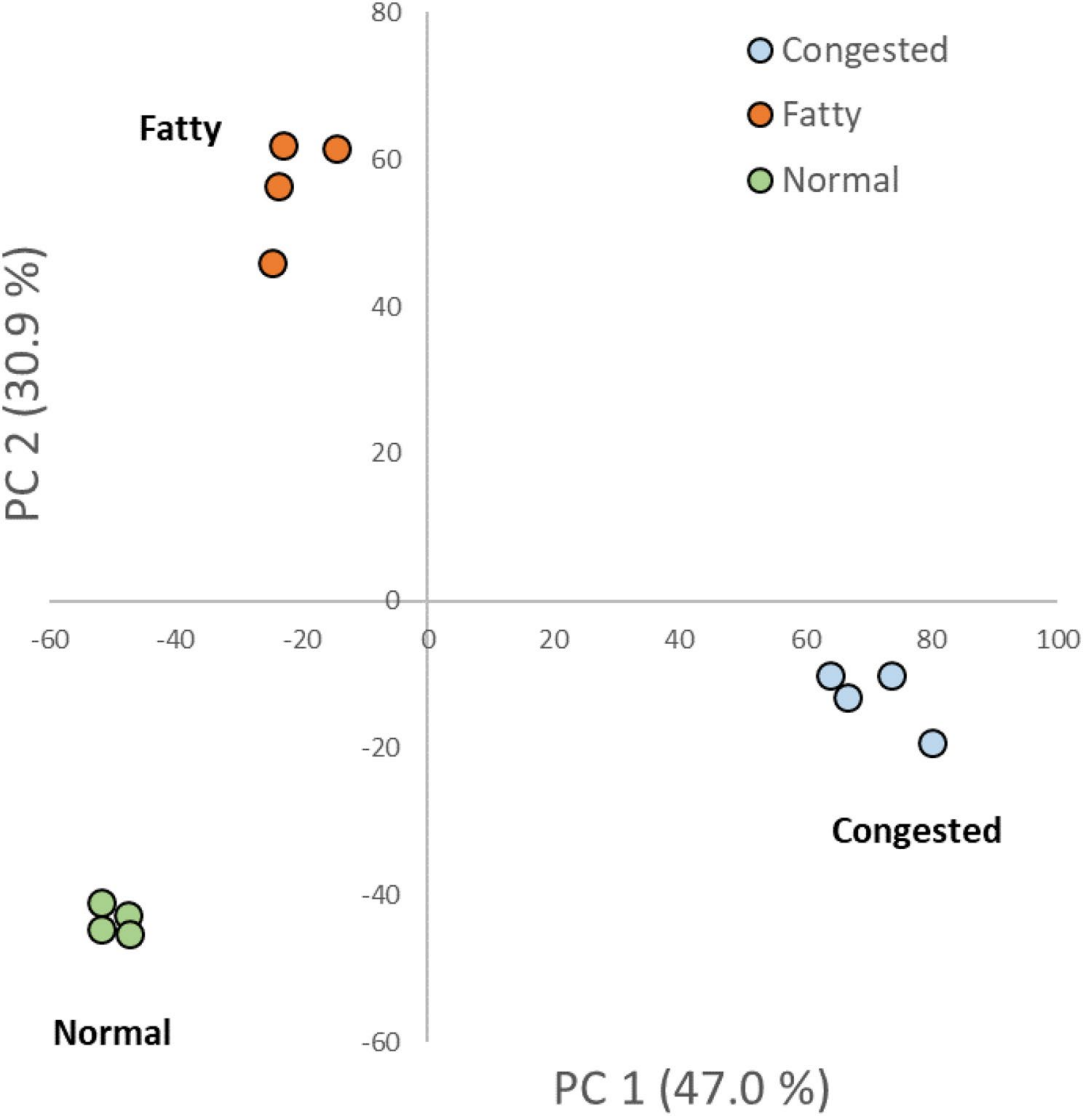
Principal component analysis (PCA) scores plot for the liver suite for differential analysis. High resolution accurate mass (HRAM) of each health state (*n* = 4) includes normal (green filled circle), fatty (orange filled circle) and congested (dark blue filled circle) liver. The values in paratheses in the axes refer to the percentage total explained variance

The primary rationale for developing RM suites with distinct metabolic profiles is to promote measurement harmonization through the detection of differences when used within and across studies. Candidate RM 8462 Frozen Human Liver Suite for Proteomics and Metabolomics is currently in development. Pathological data and body mass index (BMI) calculations were used to categorize into three cohort liver materials: Normal Liver, Congested Liver and Fatty Liver (Fig. 2). The RM will be characterized for differential expression of lipids, metabolites, and proteins. It should be highly valuable in determination of definitive levels of analytes for quality control in differential based studies. Use of such suites may help to ensure that actual differences can be detected between sample groups regardless of the instrumentation, statistical approaches, and software tools.

Candidate RM 8461 Human Liver for Proteomics is another cryogenically homogenized and freeze-dried liver tissue originally developed as a qualitative material for complex bottom-up LC–MS proteomic analysis (Davis et al., 2019c). However, RM 8461 has been assessed as a stand-alone untargeted metabolomics material (Davis et al., 2019a, 2019b) and should be a good candidate for use as the only currently available tissue-based control material in the field. Preliminary LC–MS data demonstrated over 12,000 features with CV ≤ 20% from an initial assessment of 4 vials with over 2000 putative annotations resulting from spectral library matches of both mzCloud and NIST20 databases (NIST, 2020).

#### Liver extracts for system suitability

The importance of evaluating the performance of analytical platforms in advance of conducting metabolomic measurements has been illuminated in the recent literature (Broadhurst et al., 2018; Dunn et al., 2011a, 2011b; Rattray et al., 2019; Viant et al., 2019). These system suitability QC samples are typically a mixture of a small number of metabolites in a solution absent of a sample matrix and at known concentrations. These metabolites can be monitored over time and acceptance criteria (peak shape, retention time, peak area/height) checked before the start of each analytical batch (see Sect. 4).

However, the metabolomics community is suffering from the lack of a common, everyday system suitability standard by which to benchmark instrument performance for untargeted MS-based approaches. NIST has developed a research grade test material (RGTM) 10,122 Metabolomics System Suitability Sample as a large quantity, biological extract from human livers which incorporates the entirety of a metabolome, resulting in an encompassing system suitability sample. The design of a tissue extract as a suitability standard eliminates the sample preparation variation observed within biological samples, while offering simplicity of use (rehydrate and inject) and analyte complexity for analysis of all metabolomics platforms. This sample will require initial benchmarking in conjunction with the currently used and defined standard mixtures described in Sect. 4. Once suitability is established, either or both samples can be evaluated in system suitability testing. In addition to the acceptance criteria mentioned above, the metrics used to establish suitability may include the number of detected features, total ion chromatogram, MS resolution (ppm error), and LC resolution of critical pairs (e.g., leucine/isoleucine).

In addition to use in evaluating platform readiness, RGTM 10,122 is presently available, and can be a QC tool in the comparability of instrument performance across batches, studies, and laboratories, such as large, multi-center collaborations.

### In-house matrix-based reference materials

Lower-cost QCRMs can be produced in-house to initially address QA/QC needs, however production and sampling variability limit their applicability and long-term use. As noted, as a significant challenge in design of RMs (Sect. 2.2), there is a recognized need for contiguous supply of stable, matrix-specific materials. As an alternative, an iterative batch averaging method (IBAT) (Gouveia et al., 2021) may be used to produce stable in-house RMs over the course of time with relatively low variance. The IBAT process reduces the production and sampling contributions to variance by creating a common source of material from which homogeneous aliquots are produced. The advantage of this method is that instead of producing a single large batch, which will have its own challenges in achieving homogeneity and longevity, the material is continuously generated over time. Aliquots from different batches are combined into a single tube of which only minor amounts are newly produced material. Each combined material captures small changes over time while having minimal variance between different IBAT iterations. This method is flexible, easily adjusted to the production throughput and applicable to any type of matrix, thus, suitable for QC applications but also to establish RMs for various metabolomes as an important component for metabolite annotation across analytical platforms, methods, and laboratories.

### Alternative matrices as future reference materials

The traditional biofluid-based materials such as plasma, serum and urine commonly used in clinical diagnostics are the most dominant type of samples used for metabolomics and lipidomics studies. However, many substitute biological matrices such as saliva, cerebrospinal fluid, mucous, bronchial lavage, breast milk and feces are being considered and subsequently investigated as alternative RMs to support (relatively) non-invasive metabolomics and lipidomics screening techniques. As this is a nascent area for metabolomics and lipidomics research, prototype RMs are either in the concept phase or in the early stages of development and thus are not readily available.

#### Saliva and mucosa

Salivary metabolomics has been mostly focused on biomarker discovery (Gardner et al., 2020), but the limited supply and production of saliva represent a significant bottleneck for using it and other non-blood biofluids as RMs in clinical research. The term “saliva” mainly has been used for the fluids produced in the oral cavity by glands, which includes whole-mouth saliva, gingival–crevicular fluid, parotid saliva, and submandibular/sublingual saliva. The collection of whole-mouth saliva is non-invasive and does not require special training. The complexity of saliva composition and its natural variability per individual (and secretion over time) is a significant driver for the development of a RM for use as a QC material for untargeted metabolomics analysis.

The evaluation of microbial-derived metabolites (Brown et al., 2019; Scott et al., 2020; Song et al., 2020) within mucosa and stroma such as hydrogen sulfide, ceramides, tryptophan and bile acid derivatives and their association with non-communicable chronic diseases (i.e., diabetes, non-alcoholic fatty liver disease, obesity, Crohn's and inflammatory bowel disease and cancer) is another emerging area in metabolomics. This evidence suggests that some of these microbial-derived metabolites can affect mucosa permeability and induce localized proinflammatory response (i.e., along the mucosa lining in the gut) or are able to enter the colonic epithelial cells (i.e., chronic systemic inflammation in obesity). Both targeted and untargeted metabolomic approaches, in conjunction with other multi-omics platforms, have been used to determine the effects of diet or dietary components in mucosa associated microbiota linked to disease and evaluate the efficacy of therapeutic strategies (Aden et al., 2019; Mars et al., 2020). The development of RMs for the harmonization of the measurements of microbial-derived metabolites associated with mucosa integrity represents a necessary first step in the identification of potential biomarkers of disease risk for both preclinical and clinical studies.

#### Breath and volatile analyses

There has been increasing interest in the use of breath analysis (also called breathomics), as the metabolome of the volatile organic compounds (VOCs) in breath can be captured in a facile non-invasive manner. Breath, and indeed VOCs from other sources (e.g., skin, wounds, bacteria, foodstuffs), are generally captured using sorbent materials due to the high vapour pressure of these chemical species, and as a consequence of their low concentrations (ppb is typical), sorbents also pre-concentrates them. Once captured on materials like (e.g.) Tenax, polydimethylsiloxane, or the Carbopack and Carboxen series, VOCs are released using thermal desorption (TD) and subsequently analysed by GC–MS (Lawal et al., 2017; Rattray et al., 2014). In addition to these off-line methods, some MS analyses can be performed directly on VOCs in the headspace using selected ion flow tube-mass spectrometry (SIFT-MS) or proton transfer reaction-mass spectrometry (PTR-MS) (Bruderer et al., 2019; Smith & Spanel, 2005). The breathomics community are investigating standardization of sampling and analysis of breath samples (Herbig & Beauchamp, 2014) and a recent study by Wilkinson and colleagues generated benchmark values for TD-GC–MS analysis of human breath samples containing peppermint-derived VOCs using data collected from several different research groups (Wilkinson et al., 2020). Due to the nature of the analysis and capture of VOCs, the RM cannot be a QCRM sample as it is technically very challenging to mix breath from different people (Broadhurst et al., 2018). Therefore, RMs will need to be comprised of standard mixtures of reference VOCs, with known vapor pressures and known chemistries so that they are absorbed by the sorbent materials used for VOC capture.

#### Breast milk and other fluids

There has been significant interest in the characterization of complex lipids in human breast milk beyond classical fatty acid compositional studies (George et al., 2018). Although human breast milk RMs do exist with certified values for organic contaminants (NIST SRM 1953 Organic Contaminants in Non-Fortified Human Milk and SRM 1954 Organic Contaminants in Fortified Human Milk), it does not appear that recent lipidomic studies have used these SRMs. There are known differences in the lipid content of human breast milk throughout the course of lactation, due to diet, health status, and depending on the time of sampling (i.e., foremilk vs. hindmilk) (Jensen, 1996). It is thus imperative that future studies consider adopting such materials for QC purposes in order to maximize the translatability of the results within this burgeoning area of infant nutrition. Likewise, other biofluids that have been used in recent lipidomic studies but do not presently have commercially-available RMs include cerebrospinal fluid (Reichl et al., 2020), synovial fluid (Leimer et al., 2017), and amniotic fluid (Cao et al., 2020) among others (Acera et al., 2019; Agatonovic-Kustrin et al., 2019; Gregory et al., 2013; Höfner et al., 2020; Nilsson et al., 2019; Yang et al., 2019).

#### Feces

Another emerging area in metabolomics is the characterization of microbial metabolites in human and other mammalian feces (aka stool) and its association with gut microbiota and metabolism biomarkers of health and disease. Both targeted and untargeted metabolomic profiling of human stool have been successfully used to identify a dysbiotic metabolic signature associated with disease and for the assessment of dietary intake in diverse communities (Jain et al., 2019; Kim et al., 2020; Lloyd-Price et al., 2019). However, relatively few studies have reported the optimization and QC of microbial metabolites from stool, likely due to the complexity and heterogeneity of stool specimens. Deda et al. (2017) illustrated that the pH and volume ratio for the fecal sample weight to extraction solvent in the sample preparation are critical to obtain more comprehensive metabolic profiles (untargeted and targeted), which are also platform dependent. In another study (Moosmang et al., 2019), different extraction methods and the effect of solvents were used to compare the variability in metabolome analysis from stool specimens; water as the extraction solvent yielded the best results in terms of coverage, number of detected features and reproducibility. A recent systematic study (Cui et al., 2020) found that nearly 70% of the systematic variation is related to the extraction solvent; freeze drying caused a relative loss of short chain fatty acids and lower reproducibility than wet methods utilizing raw fecal slurry stool material. Volatile metabolites in collected stool specimens present a serious challenge for preservation and require development of standardized protocols for extraction (Bosch et al., 2018). Since freeze-drying may have a strong influence on metabolite degradation, QCRMs are needed to measure the efficacy of extraction and integrity of any novel approaches used for compound preservation.

Currently, the Gut Microbiome Committee of the Institute for the Advancement of Food and Nutrition Sciences (IAFNS; formerly the International Life Sciences Institute) aims to identify and eventually quantify gut microbial metabolites that have been linked to diet and health. Following a October 2019 workshop (Mandal et al., 2020), NIST and IAFNS are now collaborating to develop a suite of human whole stool RMs to validate both metagenomics measurements associated with Fecal Microbiome Transplants (FMTs) and other live biotherapeutic products as well as metabolomic measurements to identify new biomarkers associated with the health of the human gut microbiome. Two pooled whole stool prototype RMs collected from vegans and omnivores has been developed for use in method harmonization and QC for next generation sequence (NGS) metagenomics and MS- and NMR-based untargeted metabolomics. These RMs have been demonstrated to be homogeneous with respect to both the microbial taxa (DNA) and key metabolites. They are being evaluated for longer-term (> 6 months) stability in addition to any differences in the aqueous and lyophilized storage conditions. They are being used as test materials in an ongoing gut microbiome metabolomics NIST interlaboratory study (NIST, 2021).

## Synthetic chemical standard mixtures

The need for high purity RMs comprised of individual chemical components as standard mixtures has increased over the past several years. These mixtures are used for performing routine metabolomic assay fitness evaluations, broad-based metabolite quantification and for construction of retention time and multi-stage MS (MS/MS) spectral libraries. Four major components for ensuring data quality in bioanalytical MS-based measurements are instrument qualification, analytical method validation, system suitability and quality control checks (Briscoe et al., 2007). Related to untargeted metabolomics, system suitability tests are commonly performed before, during and after an experiment to assess the system performance using a set of standard mixtures of compounds, which are either natural or stable isotope labeled compounds. In theory, the standard mixture(s) should be adaptable to a variety of metabolomic methods and applicable to different analytical workflows, whilst satisfying the aims necessary for qualification and ideally quantification. A control chart of results can reveal gas chromatography (GC), LC or MS system performance deficits (e.g., signal drift or offset, peak tailing or splitting, column degradation, mass calibration) and can alert the user to corrective maintenance that would be necessary prior to the measurement of precious experimental samples. QC check standards, comprised of an experimental sample spiked with a known concentration of stable isotope-labeled internal standards, are then incorporated into the analysis run and are designed to monitor the data quality of each sample. By establishing acceptance criteria and requirements for use as an internal standard, both qualification and quantification are inherently possible.

### In-house standard mixtures

Synthetic mixtures of chemical reference standards prepared within individual laboratories (“in-house” standard mixtures) can provide a fit-for-purpose solution to the immediate and often unique needs of untargeted metabolomics and lipidomics studies, or where RMs for specific sample types are not available. These mixtures do have limitations but are affordable and can be applied for different QA/QC processes once they have been shown to be fit for purpose. The construction of in-house mixtures generally leverages the wide variety of neat chemical standards available to tailor mixtures that meet the specific needs of the locally employed technologies and methods. Table 2 describes a variety of standard mixtures used in metabolic profiling and clearly illustrates the diversity of applications, methods, and purposes. A review of the relevant literature suggests that synthetic mixtures themselves broadly fit one of two types: (1) those intended to be used on their own (generally constituting unlabeled materials) and (2) those intended to be added to biological sample material (generally constituting stable-isotope labelled materials). These mixtures appear to be used broadly in one or more of three main contexts: (1) assurance of system suitability before, during and after a metabolomics experiment, (2) QC during an analysis, and/or (3) correction of data after an analysis. Specific applications include the monitoring of chromatographic retention time accuracy and/or precision, monitoring signal intensity accuracy and/or precision, controlling preparative or technical processes (e.g., extraction efficiency, injection volume) and/or correcting after acquisition of data.

**Table 2:** Examples of synthetic chemical standard mixtures

Mixture composition (no. of components)	Mixture type		Purpose	Application	Application context			Performance checks				
	Un-labeled	Labeled			Before	During	After	RT	Signal Intensity	*m/z*	Yield	References
Amino acids, bile acids, sugars, organic acids (7)	Y		System suitability	Run separately	Y		Y	Y	Y	Y		(Zelena et al., 2009)
Small polar metabolites (8)	Y		Quantification	Spike in sample		Y		Y	Y	Y	Y	(Zelena et al., 2009)
Small molecule metabolites, nonpolar species (13)		Y	RI QC (for POS)	Spike in sample		Y		Y	Y	Y	Y	(Evans et al., 2009)
Amino acid metabolites, organic acids (11)		Y	RI QC (for NEG)	Spike in sample		Y		Y	Y	Y	Y	(Evans et al., 2009)
Small molecule metabolites, bile acids, organic acids (14)	Y		System suitability	Run separately	Y	Y	Y	Y	Y	Y		(Gika et al., 2016)
Small molecule metabolites, organic acids (4)	Y		System suitability (for POS)	Run separately	Y	Y	Y	Y	Y	Y		(Gika et al., 2016)
Small molecule metabolite, sugar, bile acids (4)	Y		System suitability (for NEG)	Run separately	Y	Y	Y	Y	Y	Y		(Gika et al., 2007)
Small molecule metabolites, organic acids (8)		Y	RP QC	Spike in sample	Y	Y	Y	Y	Y	Y		(Lewis et al., 2016)
Amino acid, small polar metabolites (6)		Y	HILIC QC	Spike in sample	Y	Y	Y	Y	Y	Y		(Lewis et al., 2016)
Amino acid, organic acid (2)		Y	Quantification for GC–MS	Spike in sample		Y		Y	Y	Y		(Lewis et al., 2016)
Sugars (2)	Y	Y	Quantification	Spike in sample	Y	Y	Y	Y	Y	Y	Y	(Papadimitropoulos et al., 2018)
Small molecule metabolites, organic acids (6)	Y		System suitability	Run separately	Y	Y	Y	Y	Y	Y		(Pandher et al., 2009)
Amino acids, bile acids, small molecule metabolites, xenobiotics (11)	Y		System suitability	Run separately	Y	Y	Y	Y	Y	Y		(Pereira et al., 2010)
Amino acids, small molecule metabolites, lipids (22)	Y		Validation data across platforms	Run separately	Y	Y	Y	Y	Y	Y		(Naz et al., 2013)
Amino acids, lipids, xenobiotics (44)	Y		System suitability	Run separately	Y	Y	Y	Y	Y	Y		(Barri et al., 2012)
amino acids, bile acids, small molecule metabolites, lipids (7)		Y	Quantification	Spike in sample		Y		Y	Y	Y		(Barri et al., 2012)
Amino acids, lipids (7)	Y		System suitability	Run separately	Y	Y	Y	Y	Y	Y		(Broadhurst et al., 2018)
Small polar metabolites (4)	Y		System suitability	Run separately	Y	Y	Y	Y	Y	Y		(Gika et al., 2012)
Amino acids, fatty acids, sugars, organic acids (11)		Y	Quantification	Spike in sample		Y		Y	Y	Y		(Dunn et al., 2011a, 2011b)
Amino acids, lipids, small polar metabolites (14)		Y	Quantification	Spike in sample		Y		Y	Y	Y	Y	(Soltow et al., 2013)

The maintenance of in-house mixtures for routine QC applications requires procurement and characterization of neat chemicals, consideration of the stability of these chemicals individually and in mixtures, rationalized mixture design, construction of SOPs for the repeatable construction of mixtures for use as routine reagents, and validation of mixture stability over the course of intended use. When prepared utilizing well-characterized pure chemicals as starting materials and adhering to a rigorous SOP, the quality of the prepared standard mixture may even meet the ISO qualifications (ISO, 2016) and be considered equivalent to a RM. While the costs of producing in-house standard mixtures is more controllable and may be minimized for a specific application, material creation and maintenance procedures can require significant laboratory resource investment (e.g., in maintaining the capability of personnel and condition of requisite measurement instruments).

Technologies supporting metabolic profiling are numerous and varied, including LC, GC, capillary electrophoresis, and NMR. Methodology can be equally varied, for example with common modes of LC separation for metabolomics applications. These modes include reversed-phase LC (RPLC), hydrophilic interaction liquid chromatography (HILIC) and ion exchange chromatography (IEC), each of which presents additional diversity in stationary and liquid phase combinations. For this reason, QC in metabolic profiling approaches is largely supported by in-house standard mixtures.

Table 2 describes standard mixtures used in metabolic profiling and illustrates the diversity of applications, methods, and purposes. A review of the relevant literature suggests that synthetic mixtures themselves broadly fit one of two types: (1) those intended to be used on their own (generally constituting unlabeled materials) and (2) those intended to be added to biological sample material (generally constituting stable-isotope labeled materials). Similarly, these mixtures appear to be used broadly in one or more of three main contexts: (1) assurance of system suitability before, during and after a metabolomics experiment, (2) QC during an analysis, and/or (3) correction of data after an analysis. Specific applications include the monitoring of chromatographic retention time accuracy and/or precision, monitoring signal intensity accuracy and/or precision, controlling preparative or technical processes (e.g. extraction efficiency, injection volume) and/or correcting after acquisition of data.

The maintenance of in-house mixtures for routine QC applications requires procurement and characterization of neat chemicals, consideration of the stability of these chemicals individually and in mixtures, rationalized mixture design, construction of SOPs for the repeatable construction of mixtures for use as routine reagents, and validation of mixture stability over the course of intended use. Although these steps do require significant laboratory resource investment (e.g., maintaining the capability of personnel, dedicated laboratory instrument usage), they have several inherent advantages over use of commercially available mixtures. The wide variety of neat chemical standards to tailor the preparation of mixtures can meet the needs of specific situations or methods and can provide better fitness for purpose for a specific technology or methodology, such as a specific mixture to match biochemical panels or to extend across broad profiling applications.

### Commercially-available standard mixtures

When available and suitable for the application, commercially-available standard mixtures can offer laboratories ready and continuous access to qualified standard mixtures that have been consistently produced with high lot-to-lot reproducibility and have well-established stability criteria. Both convenience and third-party accreditation may be important in some applications including those in more regulated environments. In addition to the technical requirements, standard mixtures are often designed to meet any number of important factors including cost minimization, control of reagent purity, ease of preparation, and chemical stability. These benefits result at the expense of convenience and third-party accreditation or guarantee of quality, which may be important in some more regulated applications. When available and suitable for the application, commercially-available standard mixtures can offer laboratories ready (and continuous) access to qualified standard mixes that have been consistently produced with high lot-to-lot reproducibility and have well-established stability criteria.

Standards such as those discussed in this section assure that there is enough reproducibility in the data that they are worth all of the time, money and effort of further data workup. This point of view is not just relevant to the isotopic standards discussed but is equally true for all of the materials discussed in this paper. One could avoid the cost of the quality control elements, but to do so puts the quality of the data at risk. Products such as QReSS, IROA, and the other standards discussed in this review provide methods to assure and enhance data quality. The understanding and correction of all instrumentation, and source-created quantitative error, the ability to sample-to-sample normalize data, and to correctly name compounds are all critical to developing the reproducible, high-quality datasets that metabolomics needs to move forward and attain the utility it should. The elements in this section, being more chemically defined than biological mixtures and yet being standard mixtures available to all researchers provide a consistent foundation by which instrumental performance, and ultimately the quality of the data, can be understood.

#### QReSS kits

**Fig. 3:**
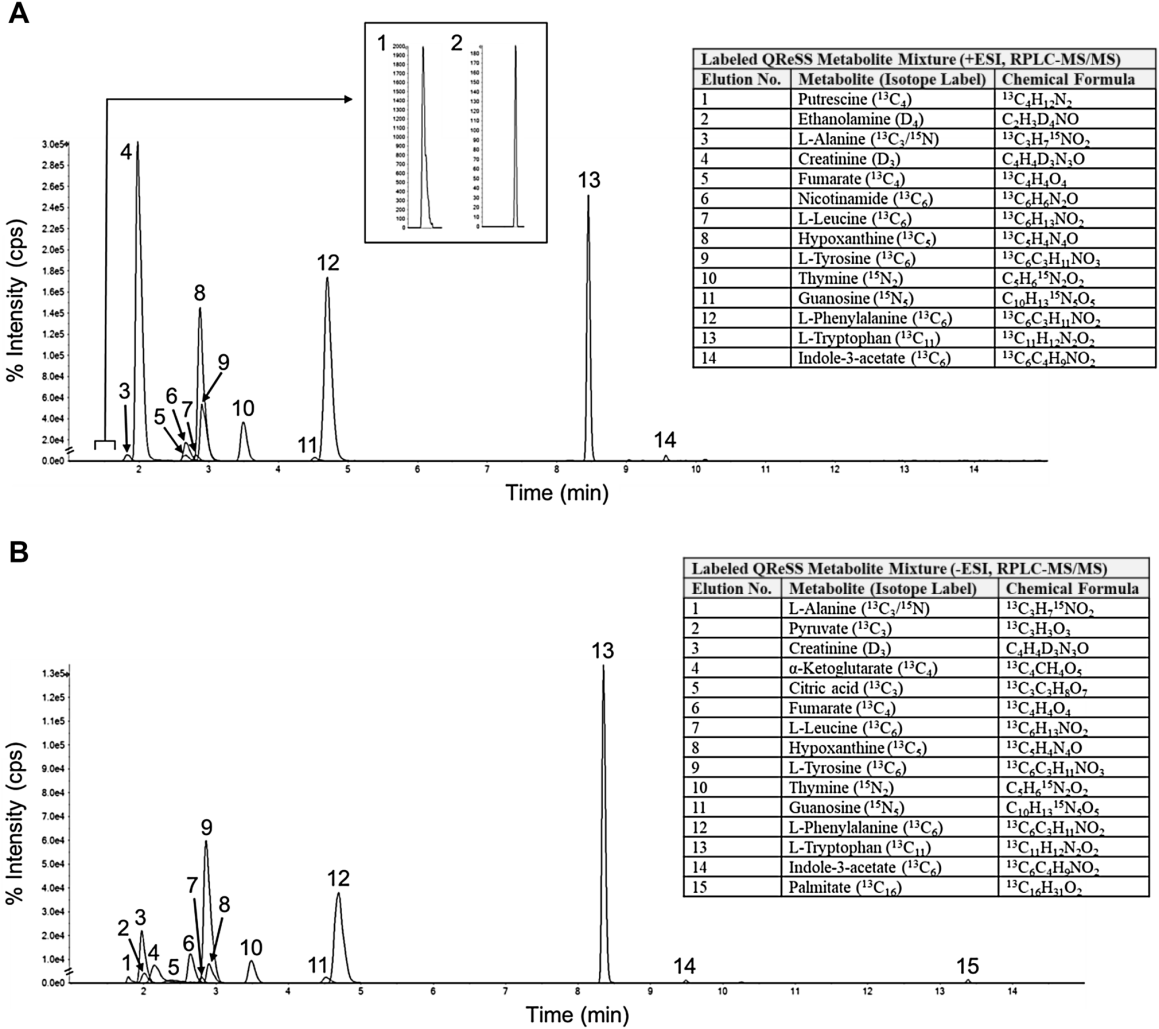
Total ion chromatogram (TIC) of a matrix-free, combined QReSS mix measured by RPLC-MS (Phenomenex Kinetex F5 column, SCIEX TripleTOF® 6600 LC–MS/MS System). Acquisition from + ESI and -ESI are shown in **A** and **B** respectively, with the annotations corresponding to the metabolite elution order in its corresponding table inset

Cambridge Isotope Laboratories (CIL) has developed multi-component standard mixes that can be applied to MS metabolomic experiments following simple solvent dissolution and mix workup. In one recent example, CIL (in collaboration with SCIEX) developed a metabolomics kit named QReSS (Quantification, Retention, and System Suitability). This kit was designed to help aid performance assessments (i.e., method QC and system suitability) and, in tandem, enable metabolite quantification in MS metabolomic workflows (untargeted, semi-targeted and targeted). This kit comprises two dried-down mixtures of 18 metabolites (in their stable isotope-labeled or unlabeled form (CIL, 2021) that span molecular weights and metabolic classes as well as chromatographic retention range. These standard mixtures are well suited for such applications due to the carefully selected compounds, inherent characteristics, and their experimental tendencies [e.g., diverse elution behavior (Fig. 3), devoid of solubility issues or stability concerns]. In one application example of system suitability with QReSS, an aliquot of the combined QReSS mixes is analyzed directly by LC–MS or LC–MS/MS using an untargeted or targeted metabolomics workflow, with performance metrics tracked over time. The nature of the MS metric tracking is predicated on the technique (e.g., targeted *vs*. untargeted), with the results ideally being displayed pictorially using Pareto plots or Shewhart control charts (González-Riano et al., 2020). Through the longitudinal monitoring of performance metrics, deviations in data quality relating to the LC and/or MS system can be illuminated and immediately addressed.

#### IROA TruQuant measurement system

**Fig. 4:**
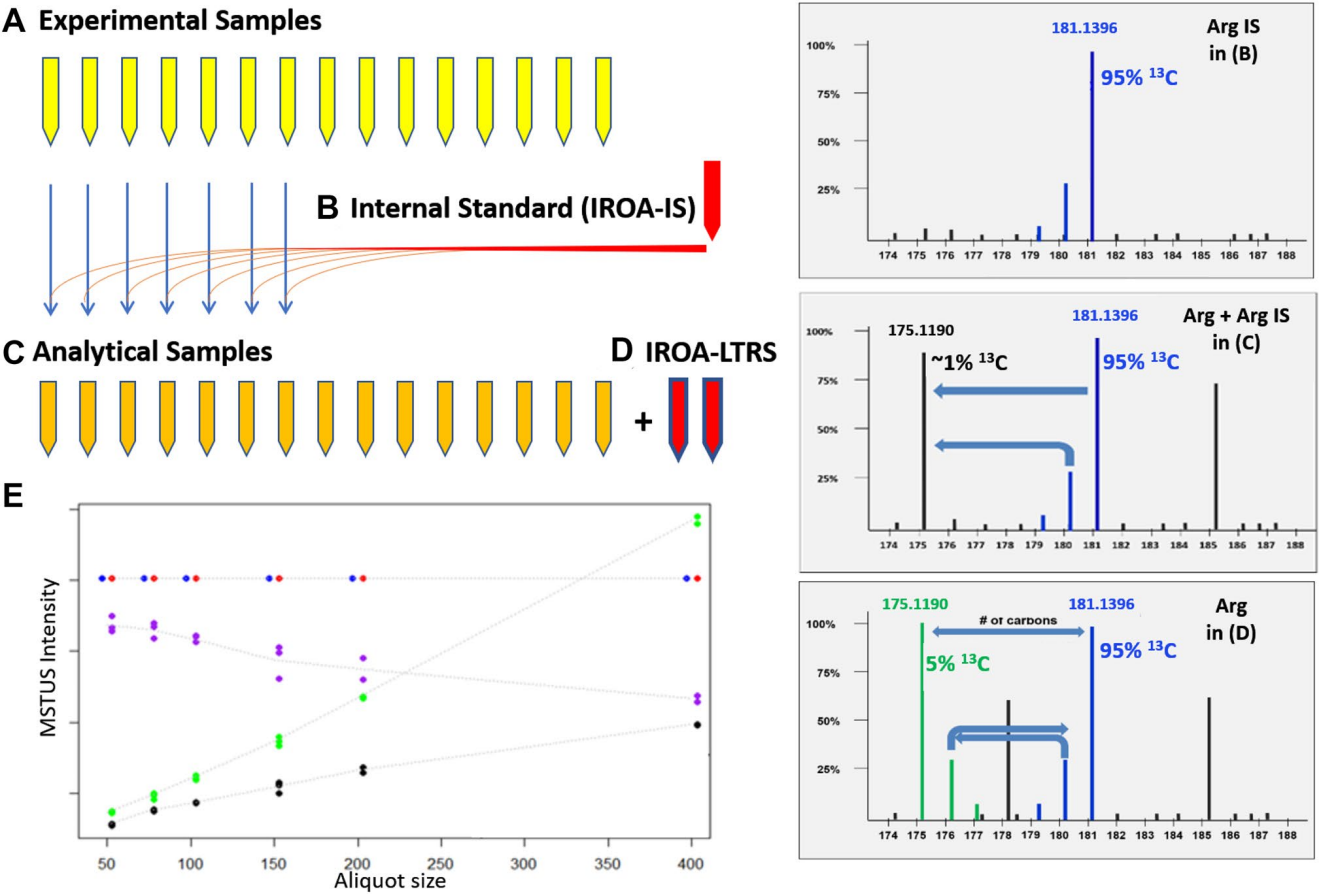
The IROA TruQuant measurement system with the experimental samples **A** is spiked with the **B** internal standard (IROA-IS) to generate **C** analytical samples that are also paired with an **D** isotopically labeled Long Term Reference Standard (IROA-LTRS). Example mass spectra of an analytical sample **C** for arginine (Arg) with the corresponding IROA-IS **B** and IROA-LTRS **D** are illustrated on the right panels. The triple-redundancy of the LTRS assures more accurate identification. An example quantification result as a normalized intensity for arginine to the IROA-IS is also provided **E**

The IROA TruQuant measurement system (see Fig. 4A–D) relies on an isotopically labeled Long Term Reference Standard (IROA-LTRS, Fig. 4D), which is paired with a chemically identical but isotopically different Internal Standard (IROA-IS, Fig. 4B). The IROA-LTRS provides a daily measure of platform QC (Evans et al., 2020), including measures of MS source function, MS instrument performance, chromatographic separation, and quantification. The IROA-LTRS is a Standard Reference Material that is run qualitatively (using data dependent fragmentation techniques) rather than quantitatively; alternate data independent fragmentation, or ion mobility scans yield validated identification of all IROA signed peaks and provide daily retention time (RT) and amplitude for all the same compounds in the IROA-IS. The IROA-IS is an internal standard that is chemically identical to the LTRS but has only the C13 isotopic (i.e., all C13 dominant isotopomers and isotopologues -Fig. 4B) which when spiked into the experimental samples (Fig. 4A) provides a mechanism for correction of ion suppression and other source-induced variances, calibration standard-based quantification, and sample-to-sample normalization for the analytical samples (Fig. 4C). The randomization of injections of the IROA-LTRS and analytical samples (containing the IROA-IS) at an injection frequency of 1–10, respectively, corrects for any within-experiment drift and provides a measure of daily instrument reproducibility if used as an injection standard, or combined sample preparation and instrument reproducibility if used as a recovery standard. Because of their biological origin these standards can be quite cost effective as injection standards and yet are quite justifiable as recovery standards because they allow for the correction of all variances imposed onto the original samples by either instrumental or preparative variability.

The IROA-IS is inserted into all analytical samples (Fig. 4C) and uses the RT and identification from the IROA-LTRS (Fig. 4D) to locate, identify, and to quantify all the natural abundance peaks associated with the IROA-IS. Quantification of metabolites is enhanced because the IROA-IS is always present at the constant concentration, and therefore (1) ion-suppression, and other source-related errors, may be corrected, and (2) the natural abundance analytical sample may be normalized to the IROA-IS (Fig. 4E; always constant). The normalization factors may be applied to all compounds in each sample, even for compounds without a specific standard.

The cost of most isotopic labels is not in the cost of the isotopic label, but rather in the cost of the isolation, and purification of individual compounds; therefore, IROA’s approach is to use standardized biologically produced compound mixtures to make the internal standards as broadly applicable as possible. In the case of the IROA IS and LTRS, the standards may be used as either recovery standards or as injection standards depending on when they are introduced, i.e., either before or after the sample preparation, with the recovery standard requiring approximately three times as much isotopic standard as the amount used as an injection standard. To put this in perspective, at current pricing, these standards cost $2 (as an Injection standard) or $6 (as a recovery standard) per sample. The “recovery cost” of most of the NIH Metabolomics Centers, and most of the academic metabolomic centers, appears to be averaging approximately $200 per sample. The major contributors to these costs are the annual instrumentation costs (including depreciation), and the personnel costs, thus the costs of the isotopic standards are running between 1 and 3 percent of the costs of the analysis. In addition, the cost of running the MS analysis is a small portion of the additional cost of the personnel time to analyze, extract, and interpret the data; all of which would be wasted if the data quality was not sufficient to justify this additional investment.

#### Standard mixtures for lipidomics

Broad-based absolute quantification is difficult in lipidomics as there are estimated to be between 10,000 and 100,000 different lipid molecular species (van Meer, 2005, van Meer, et al., 2008, Yetukuri et al., 2008; Wenk, 2010). As only a small number of commercially available isotope-labelled standards are available, it is necessary to assume that the behavior of these standards is representative for each lipid class, with respect to extraction recovery, matrix effect and mass spectrometric detection. New internal standards mixtures that could qualitatively and quantitatively represent the endogenous lipid classes distribution in plasma have been added to the catalogue of the major manufacturers, such as Avanti Lipids. The series of SPLASH® LIPIDOMIX®, tailored on either human or mouse plasma, is produced with varying amounts of labelled standards (one standard per class) and premixed in organic solvent, which can be added to a defined amount of sample prior to lipid extraction. The mixtures aid relative quantification using HILIC or direct infusion-based methods, since all the members of each class co-elute hence the matrix suppression effect is comparable. Because of this relative quantification approach, samples employing this standard can only be compared for relative differences. The new, more diverse lipid standard mixture, UltimateSPLASH, includes 69 deuterated lipids covering multiple classes with the inclusion of several molecular species (3–9) for each class. These deuterated standards can better mimic the structural characteristics of the endogenous analytes that affect their signal intensity and thus result in more accurate quantification of lipids.

Lipid subclass-specific components of the UltimateSPLASH mix have been recently made commercially available, including, for example, a phosphatidylcholine mix of five mass-labeled standards across varying fatty acyl-chain lengths and degrees of unsaturation at different concentrations. Application-specific standard mixes within the SPLASH series have also begun to be developed, such as the OxysterolSPLASH mixture, which contains 13 deuterated oxysterol standards. This standard mix is particularly interesting as it enables a bridge to be drawn between a relatively niche area within lipid research (targeted or semi-targeted sterol analyses) and the vast majority of nontargeted lipidomics research. Many of these sterol analyses are based on GC–MS and require analyte derivatization [e.g., oxysterols (Dias et al., 2018)], in contrast to the majority of the lipidomics analyses which primarily utilize LC–MS or direct infusion (shotgun) MS approaches with nonderivatized samples. The implementation of different analyte handling and preparation techniques as well as the intrinsic differences between complementary technologies and platforms has important implications for ensuring QC. The potential for further community-wide efforts, such as interlaboratory studies for GC and LC–MS-based lipid analyses, should continue to be explored, similar to what has previously been achieved in GC–MS-based metabolomics (Lin et al., 2020) and fatty acid methyl ester analysis (Metherel et al., 2019; Schantz et al., 2016). The creation of novel labeled standards that are designed for specific lipid classes, matrices and/or applications will facilitate future harmonization studies through more accurate quantification.

#### Lipidyzer™ kits

The Lipidyzer™ platform uses an expanded set of internal standards, containing over 50 deuterium-labeled lipid molecular species across 13 lipid classes to mimic the biochemistry found in human plasma. The standards were developed by SCIEX, a mass spectrometry vendor in collaboration with Avanti Polar Lipids and Metabolon, a service provider in the metabolomics industry. This approach normalizes the quantitative bias that occurs across lipids with different chain lengths and degrees of unsaturation to allow for more accurate measurements. In addition to the labelled internal standards, additional kits are available which allow a user to assess the reproducibility and sensitivity of their system before running samples. The System Suitability kit enables the user to assess the sensitivity of the assay and the reproducibility (robustness) of the platform. The SelexION® Technology Tuning kit allows the automated optimization of the differential mobility spectrometry (DMS) cell which aids in definitive lipid identification. Finally, the QC Spike Standards kit, which contains unlabeled molecular lipid species, can be added to the QC control plasma at a known concentration and monitored throughout the analysis as a QC sample. Even though these standards were developed using the above technology, the Lipidyzer Platform has since been discontinued. The internal standards are still commercially available and can be applied to any mass spectrometry platform and ion mobililty technology such as FAIMS (Thermofisher Scientific), SLIM (MOBILion Systems Inc.), etc.

**Fig. 5:**
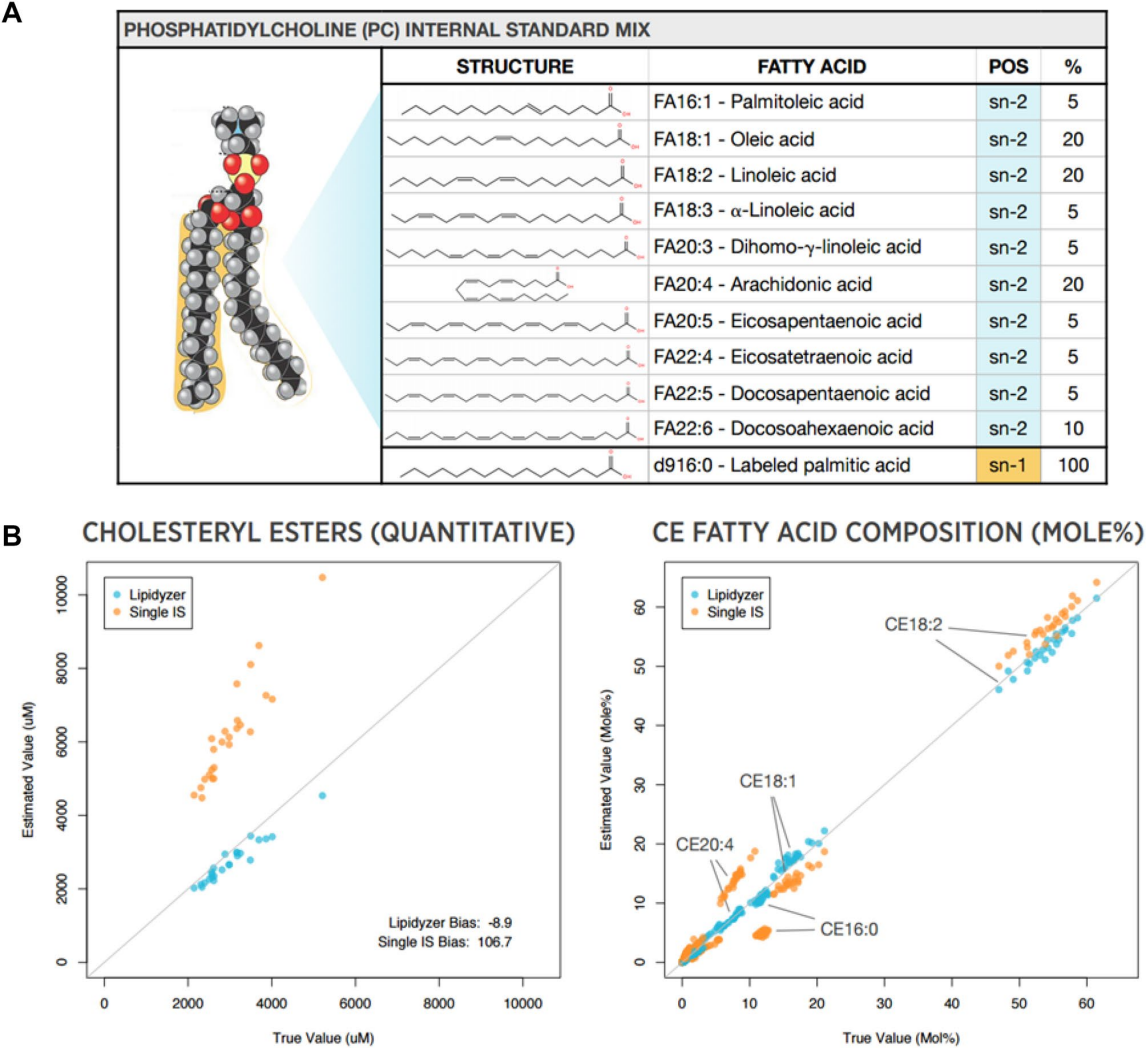
**A** An example of the Lipidyzer phosphatidylcholine (PC) internal lipid class labeling strategy. The *sn*-1 (top carbon of glycerol backbone) stereospecific numbering position is a labeled palmitate and then the *sn*-2 (middle carbon) position is changed for every fatty acid from a short chain palmitoleic acid to a long chain docosahexaenoic acid. Therefore, there are multiple internal standards to reflect the diversity of the lipid molecular species. The remainder of the 12 lipids classes have a similar strategy. **B** The Lipidyzer™ internal standards (yellow filled circle) were compared to the use of a single internal standards (blue filled circle) for their ability to accurately calibrate the concentration (μM) of total cholesteryl esters (CE) in human serum (left panel). The estimated value (using the current lipidyzer platform) versus true value (known, historical concentrations using an orthogonal LC–MS/MS platform) of the fatty acid composition of cholesteryl esters expressed as mole% fatty acid composition in human serum is also illustrated (right panel)

An example of the internal strategy used for the phosphatidylcholines (PC) class is provided in Fig. 5A. The *sn*-1 (top carbon of glycerol backbone) stereospecific numbering position is a labeled palmitate and then the *sn*-2 (middle carbon) position is changed for every fatty acid from a short chain palmitoleic acid to a long chain docosahexaenoic acid. Therefore, there are multiple internal standards to reflect the diversity of the lipid molecular species. The remainder of the 12 lipids classes have a similar strategy.

An example of the quantification data produced by the Lipidyzer™ platform is provided in Fig. 5B. Twenty-five human serum samples with known total cholesterol esters (CE) and CE fatty acid compositions were profiled using the Lipidyzer™ Platform (Ubhi, 2018). Figure 5B (left panel) highlights the Lipidyzer™ Platform quantified total CE with less than 10% bias, compared to 100% bias in the estimate made using a single internal standard. The results indicated that using a single internal standard greatly overestimated the concentration of CE, likely by overestimating the contribution of the major unsaturated fatty acids. Figure 5B (right panel) illustrates the individual fatty acid profiles of CE (expressed as a mole % of total CE) when quantified using the Lipidyzer™ Platform and the single internal standard. A recent study (Contrepois et al., 2018) utilized these standards for a comparative untargeted vs targeted lipidomics approach.

#### Biocrates absoluteIDQ

Biocrates incorporates a standard mixture of internal standards as part of a method for targeted quantification of both hydrophilic and lipid metabolites. The AbsoluteIDQ p180 kit is the most commonly used and widely adopted by laboratories conducting targeted quantification studies. It comprises analysis of 180 metabolites, mainly amino acids, biogenic amines, and lipids. The Biocrates AbsoluteIDQ p400HR kit offers a ready-to-use, standardized approach for broad lipid and metabolic profiling based on high resolution mass spectrometers, either by LC–MS data acquisition (amino acids and biogenic amines) or flow injection analysis methods (FIA-MS) (lipids). It provides quantitative or semiquantitative information for over 400 metabolites from 11 analyte groups: amino acids, biogenic amines, acylcarnitines, monosaccharides (hexose), diglycerides, triglycerides, lysophosphatidylcholines, phosphatidylcholines, sphingomyelins, ceramides, and cholesteryl esters. All kits contain calibration standards, internal standards, QC samples, and system suitability test mixes and an SOP with detailed instructions for sample preparation, instrument setup, system suitability testing, and data analysis. While the Biocrates kits are primarily designed to enable quantification, they may also be leveraged for use as reference standards for QC and chemical identification purposes in untargeted methods.

An international ring trial, with data collected by 14 laboratories, was established to evaluate the intra- and inter-laboratory precision and accuracy of the AbsoluteIDQ p400HR kit (Thompson et al., 2019) using plasma test materials from humans and rodents and NIST SRM 1950 as QC material. System suitability testing was performed prior to sample analysis, and 41 analytes in the LC–MS test mix and 17 analytes in the FIA-MS test mix were used to evaluate instrument performance, including signal abundance, mass accuracy, retention time, and peak shapes. As anticipated, both the intra- and inter-laboratory variance measured in this ring trial were far less that the biological variance observed across the study samples. Intra-laboratory variance was low for all analytes ranging from 5 to 15%, whereas interlaboratory variance across laboratories was analyte class-dependent (amino acids, cholesteryl esters, sphingolipids, and total hexoses were below 20% median CV; biogenic amines, glycerolipids, and glycerophospholipids were below 25% median CV and acylcarnitines had a median CV of 38%). Ultimately, the AbsoluteIDQ p400HR ring trial demonstrated that through system suitability testing, SOPs, and RMs, reproducible quantitative metabolomics data could be obtained across different instruments and laboratories. The performance of specific lipidomics platforms was also explored by Siskos et al. (Siskos et al., 2017) in which six different laboratories measured shared materials including SRM 1950 as a harmonization RM with AbsoluteIDQ p180 Biocrates kit. Likewise, most metabolites were observed with interlaboratory variance of below 20% CV (median).

## Reference libraries and other data harmonization approaches

As described in Sect. 3, biological RMs applied in metabolomic and lipidomic applications contain hundreds or thousands of metabolites present in a complex matrix that are sourced from a range of biological subjects. The mass concentration and chemical identity of some or all metabolites are not known, and for some molecules the stability is also undetermined. As an alternative, reference libraries (RLs) comprised of more limited number of specific metabolites or lipids as reference standards or standard mixtures can be applied that are present and/or detectable in various biological RMs. As synthetically prepared solutions or neat standards, they can also be spiked into biological matrices prior to analysis. These RLs can be applied in different approaches including to aid metabolite quantification, to apply as system suitability test samples or to construct retention time and MS/MS mass spectral libraries for metabolite identification. Some RLs offer library-specific software to find peaks and simplify library construction. A summary of RLs and related standard mixtures currently available is provided in Table 3.

**Table 3:** Examples of commercially-available reference libraries and related standards

Reference library/standard mixes	Manufacturer/supplier (product code)	Composition	QC purpose
Glycolysis/gluconeogenesis metabolite library	MilliporeSigma (ML0013)	10 mg Each of 18 glycolysis and gluconeogenesis pathway metabolite neat standards	Chemical identification
Amino acid standard	MilliporeSigma (AAS18)	(1.25–2.5) μM solutions (1 mL) of 17 amino acids	Chemical Identification/system Suitability
Cell free amino acid mixture—13C,15 N	MilliporeSigma (767,964)	(5–100) mM solutions (1 mL) of 20 stable isotope-labeled amino acids	Chemical identification
Mass spectrometry metabolite library	MilliporeSigma/IROA technologies (MSMLS)	600 Metabolites (inc. carboxylic acids, amino acids, biogenic amines, polyamines, nucleotides, coenzymes, vitamins, mono/disaccharides, fatty acids, lipids, steroids, hormones) in 96-well format	Chemical identification
QRESS standard kit	Cambridge isotope laboratories, Inc./SCIEX (MSK-QReSS)	Lyophilized for reconstitution to (2 to 100) μg/mL of 18 stable isotope-labeled metabolites. Companion unlabeled mix separately available	Chemical identification/system suitability and quantification
Amino acid standard mixes	Cambridge isotope laboratories, Inc. (e.g., MSK-CAA)	Lyophilized for reconstitution to (1.25 to 2.5) mM of 20 canonical, stable isotope-labeled amino acids. Companion unlabeled mix separately available	Chemical identification/system suitability and quantification
Organic acid standard mixes	Cambridge isotope laboratories, Inc. (MSK-OA)	Lyophilized for reconstitution to 250 mM of 33 stable isotope-labeled organic acids. Companion unlabeled mix separately available	Chemical identification/system suitability and quantification
Bile acid standard mixes	Cambridge isotope laboratories, Inc. (MSK-BA)	Lyophilized for reconstitution to 100 mM of 16 (6 unconjugated and 10 conjugated) stable isotope-labeled bile acids. Companion unlabeled mix separately available	Chemical identification/system suitability and quantification
SPLASH LIPIDOMIX® Mass spec standard	Avanti polar lipids (330,707)	(2 to 350) μg/mL solutions of 14 lipids	Chemical identification/system suitability/quantification
Human Metabolites V12.0	MetaSci (https://www.metasci.ca/products)	5–10 mg of 1200 metabolites (inc. organic acids, fatty acids and esters, vitamins and co-factors, purine metabolism, amino acids, food additives and components, bacterial (E. coli), plant and fecal sources)	Chemical identification
AbsoluteIDQ p180 and p400 and Quant 500 Kits	Biocrates (MxP, AbsoluteIDQ)	Patented 96-well plate with internal standards, calibrators, QCs and system suitability tests. SOP included as well as software for data processing and interpretation	Chemical Identification and quantification
Lipidyzer Kits	SCIEX (XXXISTLPV-100)	Internal Standards Kit (over 50 labeled standards), SelexION® Tuning Kit (for ion mobility), System Suitability Kit, and QC Spike Standards with Control Plasma Kit (reference material). SOP included	Chemical identification/system suitability and quantification

RLs comprised of standard samples or mixtures are constructed with authenticated chemical standards representing naturally occurring metabolites or lipids. RLs can focus on a specific metabolic pathways or processes (e.g., glycolysis/gluconeogenesis kits) or metabolite classes (e.g., amino acids, organic acids, lipids, bile acids), or compounds from more general primary metabolism. These products distributed as individual standards or kits are also provided in Table 3. While some RLs are designed for targeted quantitative applications, all are leveraged by the untargeted metabolomics and lipidomics communities to generate suitable databases for metabolite identification and cross site comparison of chromatographic retention times and reference mass spectra. These authentic compound libraries may be found both as natural abundance and isotopic libraries, depending on need.

Apart from RMs developed specifically for metabolomics, other chemical property standards of wider application may assist metabolomics researchers to improve reporting and harmonization of method and results. As the retention time of analytes is not molecule specific, but instead it is highly dependent on system conditions, reporting retention indices (RIs) have been used as a gold standard in GC analysis for some decades to correct for instrument or column variability. In metabolomics applications, the GC–MS Metabolomics RTL Library (Kind et al., 2009) incorporates RIs based on the use of fatty acid methyl esters (FAMES). Recently the National Research Council Canada developed a reference material (RM-RILC) (NRC, 2021) designed for the measurement of LC RIs based on 20 homologous *N*-alkylpyridium-3 sulfonates in methanol that bear increasing hydrophobicity. Its use in interlaboratory study of five different LC–MS systems was shown to minimize relative deviation and improve cross laboratory comparisons (Quilliam et al., 2015).

Similar developments have been independently reported in untargeted LC–MS analysis from the NORMAN network of reference laboratories (NORMAN, 2021) for monitoring of emerging environmental substances. Overall, analysis of such standards and calculation of RIs for known analytes and for recurrent unknowns shows promise to help method validation, improve comparison of data obtained in different laboratories and thus harmonize reporting, but further collaborative interlaboratory work is needed. Similarly, the EPA’s non-targeted analysis (NTA) collaborative trial (ENTACT) (Ulrich et al., 2019) was conducted with the aim to move the untargeted analysis community towards an improved comparison of methods and results, and ultimately to devise performance benchmark standards. The recent formation of the Benchmarking and Publications for Non-Targeted Analysis (BP4NTA) Working Group (BP4NTA, 2021) aims to motivate the NTA community toward competency for proficiency testing. Furthermore, significant challenges exist to reconcile the inconsistencies in compound identification that result from incompleteness of databases and varied results from different data pipelines. The successes (and failures) of the standardization efforts within these corresponding NTA communities may be leveraged and implemented into practice within metabolomics and lipidomics communities.

## Implementation and outreach

In addition to publication efforts, the mQACC is committed to promote, highlight and disseminate the needs and utility of RMs to the broader metabolomics community through leadership and active participation in interlaboratory studies, cooperation with other consortia, and engagement in symposia and workshops at relevant society meetings and international conferences. The mQACC will also seek to generate online tutorials and videos. With respect to social media, mQACC has a twitter account (https://twitter.com/mQACC) which we shall also use to highlight advances in standards for quality assurance and quality control, disseminate important QA/QC scientific publications and make recommendations on best practices from mQACC to the metabolomics community.

### Interlaboratory studies

Often referred to as ring trials and round-robin comparisons, interlaboratory studies are useful in allowing metabolomics and lipidomics researchers to assess differences and validate their measurement processes and methodology. Similar to proficiency testing schemes, interlaboratory studies can be used to demonstrate measurement competency, which can be advantageous for QA applications of qualifying recently trained personnel and ensure that a new method performs as anticipated. More importantly, interlaboratory studies that utilize RMs and other QC materials are an effective tool to determine sources of variation or challenges that impact metabolomics measurements and untargeted profiling efforts.

In recent years, several interlaboratory studies have been conducted with SRM 1950 as a QCRM but has been applied for largely targeted metabolomics and lipidomics (Bowden et al., 2017; Cheema et al., 2015; Siskos et al., 2017; Thompson et al., 2019). While designed to define metabolite specific (targeted) reproducibility and accuracy, the results can also inform good laboratory practices that benefit untargeted analysis techniques. Interlaboratory studies have been launched recently (2019) and in cooperation with the mQACC as part of NIST’s MetQual program. The primary goals for the interlaboratory study were: (1) to support measurement comparability of untargeted metabolomic profiling in human plasma, (2) assess measurement variability within the untargeted metabolomics community, (3) to evaluate and ascertain a (qualitative) consensus characterization of metabolites present in the candidate RM 8231 plasma suite, and (4) to gather feedback from the untargeted metabolomics community on the potential functions and implementation practicality (fit-for-purpose) nature of the RM suite. Participants agreed to measure metabolomic profiles using the routine metabolomics sample preparation protocols and analytical methods and data acquisition employed by their labs, in addition to using their customary data processing, data curation, and multivariate analysis methodology. The collection of results from the participants and the resultant analysis of the data is ongoing.

Early community-wide harmonization efforts through interlaboratory studies in lipidomics were focused more on broadly defining the lipidome and less on a centralized or accepted workflow (Bowden et al., 2017; Quehenberger et al., 2010). More recently, the Reference Material and Biological Reference Ranges interest group of the International Lipidomics Society (ILS), in collaboration with NIST and Avanti Lipids, launched in January 2020 a series of international ring trials, each one focused on measuring the absolute concentration of different selected lipid species of clinical relevance in RMs. Initially, SRM 1950 and the suite of NIST plasma RMs described in Sect. 3.2 were distributed to approximately 40 laboratories around the world (located in Europe, the Americas, Asia and Australia) that were asked to quantify four ceramides (Cer d18:1/16:0, d18:1/18:0, d18:1/24:0 and d18:1/24:1), based on their clinical utility (PMID: 27,125,947) in the cardiovascular disease field. The first phase of the trials is centered on RMs to establish a global network of laboratories that will then collect samples from different human cohorts in healthy and diseased conditions and measure the same lipids that were quantified previously in RMs. The initial RM evaluation is anticipated to enumerate interlaboratory differences, which provides a critical first step to provide confidence in lipid concentration reference ranges from cohort data and thus drive translation towards more clinical applications. Even though these are designed and evaluated as targeted interlaboratory studies, the aim is to pinpoint specific aspects of workflows that cause non-agreement in community results, which will ultimately improve untargeted lipidomics applications.

In addition to the use of common RMs across metabolomics and lipidomics laboratories, the knowledge gained by these community-based activities can foster the development of best practice guidelines, and ultimately reduce measurement variance within metabolomics and lipidomics. Given the foremost goal of clinical translation of scientific findings, these efforts are anticipated to significantly reduce measurement variance while improving community measurement agreement, thus facilitating the fields of metabolomics and lipidomics to reach their full potential of producing results of clinical significance.

### Stakeholder outreach

A significant driver is also to inform stakeholders and provide educational outreach on current and prospective RMs and associated QA/QC tools available for safeguarding untargeted metabolomics and lipidomics measurements. This effort is aimed at both newcomers as well as experienced analysts and researchers. For example, mQACC members that are also a part of the ILS Reference Materials and Biological Reference Ranges working group engage and support the researchers in this field to implement the use of RMs as common practice. The initial effort is focused on the correct use of SRM 1950 and the new plasma RM suite described previously; further activities will promote the characterization of other materials, such as urine or specific lipid classes (e.g., bile acids in stools, triacylglycerol in liver). Corresponding guidelines for standardization, method validation and reporting of lipid molecules are currently being developed within ILS as community-wide effort (Liebisch et al., 2019). The development and implementation of novel RMs with matrix-specific lipidomic profiles and the identification of novel lipids will also have important implications in agriculture and nutrition applications.

Other stakeholder clinical organizations and working groups such as the International Federation of Clinical Chemistry (IFCC) Scientific Division also support the development of RMs and reference measurement procedures that are traceable to these materials to promote standardization in laboratory medicine. There is a keen interest in RMs that can be easily integrated into clinical application workflows to allow for reliable clinical biomarker measurements. However, the development of appropriate RMs for clinical applications requires the collaboration of clinical researchers in the field of untargeted metabolomics to ensure their suitability in clinical practice. Because differential metabolite and lipid signatures can be caused by a disease state, there may be some interest in the development of RMs that represent the pooled serum and/or plasma from chronic disease patients (i.e., patients suffering from autoimmune diseases, diabetes, cancer, kidney disease, cardiovascular disease, etc.). A defined or standardized biological matrix in the form of a commutable RM will also aid in finding biomarker signatures to help elucidate clinical questions and provide reliability in clinical measurements established methods (Dias and Koal, 2016). The issue of commutability (or equivalence through an established method) between the applied matrix-matched RM to the representative metabolomics test samples always remains.

Lastly and equally as important, mass spectrometry vendor and academic research organizations who are at the forefront of developing and innovating new technology and applications have a major stake in the implementation of reference materials, reference standards and system suitability test mixtures. These organizations utilize such materials and products for standardization of the technology and methodologies, as well as validation and verification at product development stages for a range of applications. Collaborative engagement between commercial reagents and consumables manufacturers and metabolomic researchers and leaders will enable technological solutions to address the many challenges described here.

The descriptions and associated references provided in this review offer a foundational knowledge on available QA/QC tools and best practices to aid the untargeted metabolomics community. Some might prefer didactic learning methods to the experiential (self-taught) approach, thus engagements with experts or hands on training may better instill the justification of the suggested practices. Such opportunities include a specific QA/QC in Metabolomics course by the University of Birmingham Training Center and topic sections within general metabolomic training courses offered by institutions such as European Bioinformatics Institute (EMBL-EBI) or the NIH Metabolomic Program Regional Comprehensive Metabolomics Resource Cores (RCMRCs). Workshops, webinars [e.g., Chemical & Engineering News webcast of a QA/QC Practices in Untargeted Metabolomics (C&EN, 2020)] and any other recorded materials also offer exposure to a broad audience and are additional opportunities for training and engagement on the appropriate usage of RMs and the current state of QA/QC practices. The mQACC Best Practices working group also began hosting a series of interactive discussion workshops (Metabolomics Association of North America (MANA) 2019, with the Human Health Exposure Analysis Resource (HHEAR), focusing on current practices of QA/QC regarding topics such as pooled QC use and system suitability testing.

## Summary

We have highlighted numerous and diverse RMs from well characterized mixtures of authentic standards of complex and less well characterized biosamples that are currently available for use, in a variety of ways, as QC tools to help ensure that metabolomic and lipidomic research is as robust and reproducible as possible. Clearly numerous challenges and barriers still exist however with the continued engagement of the metabolomics community, the development of new RMs, commercially available chemical standards and reference data products will help to address them. This will require the adoption of agreed methods for their use, supplemented through well-designed SOPs which will further promote sound QA/QC practices.

## Conclusions

RM’s, whether libraries of standards, mixtures or biologically based material represent a useful source of materials that can be used to calibrate, standardize, and compare the results obtained by different laboratories. Whilst they do not represent total solutions to the many problems posed by the attempt to use untargeted metabolic phenotyping for the comprehensive characterization of the metabolomes of all of the systems being studied they nevertheless represent an important tool in the armament of the analyst undertake such work. Here we have reviewed the currently available materials and indicated some of their applications.
